# Gaussian process models for geographic controls in phylogenetic trees

**DOI:** 10.12688/openreseurope.15490.1

**Published:** 2023-04-17

**Authors:** Frederik Hartmann, Gerhard Jäger

**Affiliations:** 1 Department of Linguistics, University of North Texas, Denton, Texas, 7620, USA; 2 Seminar für Sprachwissenschaft, University of Tübingen, Tübingen, Baden-Württemberg, 72074, Germany

**Keywords:** Phylogenetics, Gaussian processes, geospatial confounding, control for language contact

## Abstract

**Background:** Geographical confounding in phylogenetic inference models has long been an issue. Often models have great difficulty detecting whether congruences or similarities between languages in phylogenetic datasets stem from common genetic descent or geographical proximity effects such as language contact.

**Methods:** In this study, we introduced a distance-based Gaussian process approach with latent phylogenetic distances that can detect potential geographic contact zones and subsequently account for geospatial biases in the resulting tree topologies.

**Results:** We found that this approach is able to determine potential high-contact areas, making it possible to calculate the strength of this influence on both the tree-level (clade support) and the language-level (pairwise distances).

**Conclusions:** Gaussian Process models are useful tools to address geographical confounding in phylogenetic analyses. The approach has advantages and disadvantages similar to comparable distance-based approaches, yet with this method, the level of confounding can be gauged and linguistic contact zones can be identified.

## Introduction

Phylogenetic linguistics started with the aim to apply phylogenetic inference algorithms from computational biology to data based on collections of cross-linguistic lexical and grammatical data. The goal of this enterprise is to infer phylogenies, i.e., tree diagrams representing the diversification history of language families. These family trees are interesting for historical linguistics in their own right, but they also provide stepping stones for investigations into deep population history, statistical control for typological studies, and several other applications (see, *inter alia*, Bouckaert *et al.*, 2012; Bowern & Atkinson, 2012; Hruschka *et al.*, 2015; Jäger & Wahle, 2021).

A well-known potential problem of phylogenetic inference algorithms in this domain is that they model language change exclusively as vertical transmission with random mutations. However, historical linguists have been well aware since the 19th century ( Schmidt, 1872) that horizontal transmission via language contact plays an important role in language change. Modeling the effects of language contact as independent random mutations potentially introduces a bias. Also, it ignores an important source of information about language history and concomittant population history processes.

This problem has not gone unnoticed. There are studies such as ( Greenhill *et al.*, 2009) assessing the robustness of phylogenetic inference. A novel approach by Neureiter *et al.* (2022) enriches phylogenies with contact edges, i.e., connections between simulatenous points on different branches of a phylogeny enabling information transfer. The phylogenetic skeleton and the contact edges are inferred simultaneously.

This approach, however, ignores an important source of information, namely the fact that language contact almost always happens under spatial proximity. It seems therefore natural to model the effect of language contact as a spatial stochastic process. Spatial statistics have recently gained popularity in cross-linguistic studies, e.g., to identify linguistic areas or as a statistical control for typological studies ( Guzmán Naranjo & Becker, 2021; Ranacher *et al.*, 2021). Phylogenetic information is either not used at all or only in a rudimentary fashion in this context though.

The present paper presents a hybrid approach combining spatial with phylogenetic modeling. Briefly put, the probability of a certain language *L* having a certain feature *f* is assumed to depend on both the presence or absence of *f* in *L*’s spatial neighbors and in *L*’s genetic relatives. Phylogenies are obtained via statistical inference while controlling for language contact. By comparing the results with vanilla phylogenetic inference, regions of intense language contact can be identified.

First, we present the model architecture and mechanics of the model before testing the model on datasets of known language families with known contact effects, namely the *Automated Similarity Judgment Program* (ASJP) database ( Wichmann *et al.*, 2016) in the modified form with cognate inferences produced by Jäger (2018) and *Northeuralex* ( Dellert *et al.*, 2020).

## Methods

### Model architecture

**Figure 1.**
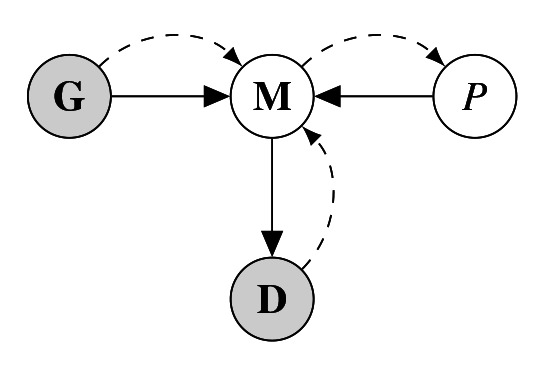
Graphical representation of the inference model. Grey nodes represent observed nodes (i.e. data) while white nodes are inferred. Black arrows indicate model-internal connections while dashed arrows show the structure of information flow in the model.

The goal of this model is to infer phylogenetic similarity between languages based on data while geographical confounds are accounted for. This can be achieved by building a model that treats phylogenetic similarity as a latent variable that is inferred as the residual similarity of two languages when linguistic similarity based on geographic proximity is accounted for. Such a model structure can be graphically visualized as the graph in Figure 1.

In this model, the statistical model **M** takes in the geographical similarity **G** and the phylogenetic similarity *P* and models those on the linguistic data **D**. Crucially, *P* is an inferred (latent) node which is the estimand in this analysis. More concretely, the model is built with **G** and **D** as inputs, whereas *P* remains in variable form. As a result, information in the model flows from the linguistic data and the geographic similarity to the model which makes it possible to infer the phylogenetic similarity. The following model architecture was used for this analysis:


$$\begin{array}{l} C_{s,l}\sim Binomial(1,p_{s,l})\\ logit(p_{s,l})=\rho_{s,l}+\gamma_{s,l} \end{array}$$

$$\begin{array}{l} \left[\begin{array}{c} \gamma_{s,l_{1}}\\ \gamma_{s,l_{2}}\\ \cdots \\ \gamma_{s,l_{n}} \end{array}\right]\sim MVNormal\;\left(\left[\begin{array}{c} 0\\ 0\\ \cdots \\ 0 \end{array}\right],\;G\right)\\ \left[\begin{array}{c} \rho_{s,l_{1}}\\ \rho_{s,l_{2}}\\ \cdots \\ \rho_{s,l_{n}} \end{array}\right]\sim MVNormal\;\left(\left[\begin{array}{c} 0\\ 0\\ \cdots \\ 0 \end{array}\right],\;P\right) \end{array}$$

$$\begin{array}{l} G_{t\;j}=\alpha_{G}e^{-\frac{\delta_{G,t\;j}^{2}}{\lambda_{G}}}\\ P_{rk}=\alpha_{P}e^{-\frac{\delta_{P,rk}}{3}}\\ \delta_{P,rk}\sim Exponential(1)\\ \alpha_{G},\alpha_{P},\lambda_{G}\sim Exponential(2) \end{array}$$

Here, the outcome *C* is a binary linguistic character of a certain language *l* and site *s*. ^1^ *C* is modelled as a binomial outcome with a probability of *p* for this specific site and language. The probability is calculated as the logit sum of the variables *ρ* and *γ*. *ρ* is the phylogenetic similarity effect to be inferred from the latent variable and *γ* is the geographic similarity effect inferred from the geographical data. Both variables are modelled as Gaussian processes, drawn from a multivariate normal distribution with a mean vector of 0 and the covariance matrices **G** and **P**. Those are themselves derived from the kernel functions that are, in the case of the geographic similarity ( *G*), based on a quadratic kernel (cf. McElreath, 2018, ch. 14.5) and the kernel of *P* is based on a Ornstein–Uhlenbeck kernel. The kernels take in the pairwise distances (phylogenetic or geographic) *δ _P_*/ *δ _G_* between two languages and output the covariance matrix of all languages. The pairwise distances for the phylogenetic kernel are drawn as a latent variable from an exponential distribution with rate 1, while the parameters for the kernels are sampled from an exponential distribution with rate 2. This results in less prior probability being allocated to high maximum covariances and high covariance values for each language pair. In effect this means that the model assumes there to be little covariance between languages *a priori*.

Note that the Ornstein–Uhlenbeck kernel has a fixed parameter *λ* = 3. This follows a modelling decision that takes into account the latent property of the phylogenetic distances: were *λ* (the magnitude of the covariance between languages) inferred, there would be more than one solution for every covariance-distance pair. In practice, the distance function $e^{-\frac{\delta }{\lambda }}$ at *δ* = 1, *λ* = 1 has the same solution (≈ 0 *.*368) as at *δ* = 2, *λ* = 2. If both parameters are inferred, for the model, both solutions are equivalent. In other words, if the model infers a covariance between two languages of 0 *.*368, it is mathematically compatible with either *δ* = 1, *λ* = 1 or *δ* = 2, *λ* = 2. ^2^ This means that if both *δ* and *λ* are inferred parameters, there cannot be a single-valued solution for any value, leading to extreme collinearity between both parameters. As a result, we fixed *λ* such that this issue is avoided.

### Data and contact area selection

As datasets on which to test the model, we used the databases *ASJP* ( Wichmann *et al.*, 2016) with cognate inferences produced by Jäger (2018) ^3^ and *Northeuralex* ( Dellert *et al.*, 2020). Both datasets are large multi-language lexical cognate databases intended for use primarily for phylogenetic inference. *Northeuralex* was specifically selected since with Dellert (2019), there exists a detailed prior study of geographical contact effects on loanwords particularly in the Baltic Sea regions. Therefore, the results of the study at hand, and thus the performance of the model, can directly be compared to a prior study.

From both datasets, a subset of languages (25 from *ASJP* and 24 from *Northeuralex*, refer to the appendix ^4^ for the full list) were selected in such a way that known high-contact areas are reflected in the dataset on which the model’s performance can be tested. Those areas are: contact between Celtic and English in the British Isles; the Balkan Sprachbund that includes South Slavic languages alongside Albanian, Romanian, and Greek; the Baltic Sea area with contact between Germanic, Slavic, Uralic, and Baltic; the contact zone between Indo-Iranian languages. As a condition for the Gaussian process approach to succeed, we expect the model to recognize these zones by increasing the geographical covariance between languages in these areas, thus increasing the likelihood for individual cognates to be borrowed between constituent languages.

The datasets were adapted to a format suited for the model architecture presented in Section *Model architecture* after conversion, the data consist of binary vectors where each vector represents a shared cognate between two or more languages. Table 1 illustrates this data shape.

**Table 1.** 

	A	B	C	D
Cognate 1	1	1	1	0
Cognate 2	0	1	0	1

Here, hypothetical languages *A*, *B*, and *C* share hypothetical cognate 1 whereas only languages *B* and *D* share cognate 2. Due to this structure, the model can calculate a probability for each language to have that cognate given the geographic and phylogenetic distances to all other languages.

## Results

We present the results of the analysis on three levels: firstly, we investigate the strength of the geographical confound between languages on a general level, before investigating the effects the geographical control has on the tree topologies of the datasets. Lastly, we look at the results on a character level, comparing the model’s inference for the likelihood of individual borrowings in the *Northeuralex* dataset with the borrowings identified in Dellert (2019).

The model was run separately on each dataset so as not to add noise to the analysis due to potentially different coding schemes. Likewise, a ‘confounded’ model was run where the geographical variable was omitted. This allows for a direct comparison between the models with and without the geographical confound.

For modelling, we used the Bayesian programming language STAN ( Stan Development Team, 2022). All results were based on posterior samples. Concretely, we extracted the posterior samples of the inferred pairwise phylogenetic distances between languages and constructed a UPGMA phylogram for each posterior sample. This yields a large number of trees from which clade support values were extracted and the consensus and maximum clade credibility (MCC) tree were constructed.

It was observed that the pairwise distances in the four-chain setup of the analysis did not achieve convergence. However, when analysing the individual chains, they yield the same tree topology. We found that this convergence issue is in part due to the fact that the same tree topology can be expressed with different distance matrices. For example, the following hypothetical upper-triangular distance matrices, *M*_1_ and *M*_2_, yield equivalent tree topologies between three hypothetical languages (not considering branch length).


$$M_{1}=\left[\begin{array}{cc} 1 & 2\\ & 1 \end{array}\right]\;M_{2}=\left[\begin{array}{cc} 2 & 4\\ & 2 \end{array}\right]$$

Here, matrix *M*_2_ is a factor of *M*_1_, meaning that the relative pairwise distances are equal. Since these distances are inferred as a latent variable in the model, each chain of the Bayesian model can converge at either *M*_1_ or *M*_2_, yielding the same tree topology but, by virtue of having converged on different values, interchain convergence is not reached. As a result, one needs to check model accuracy based on the tree topologies obtained from posterior samples directly. As described above, the different sampling chains of the models yielded tree topologies with minor differences in topology and clade support (see discussion in the appendix ( Hartmann & Jäger, 2023).

### Evaluating the model

**Figure 2.**
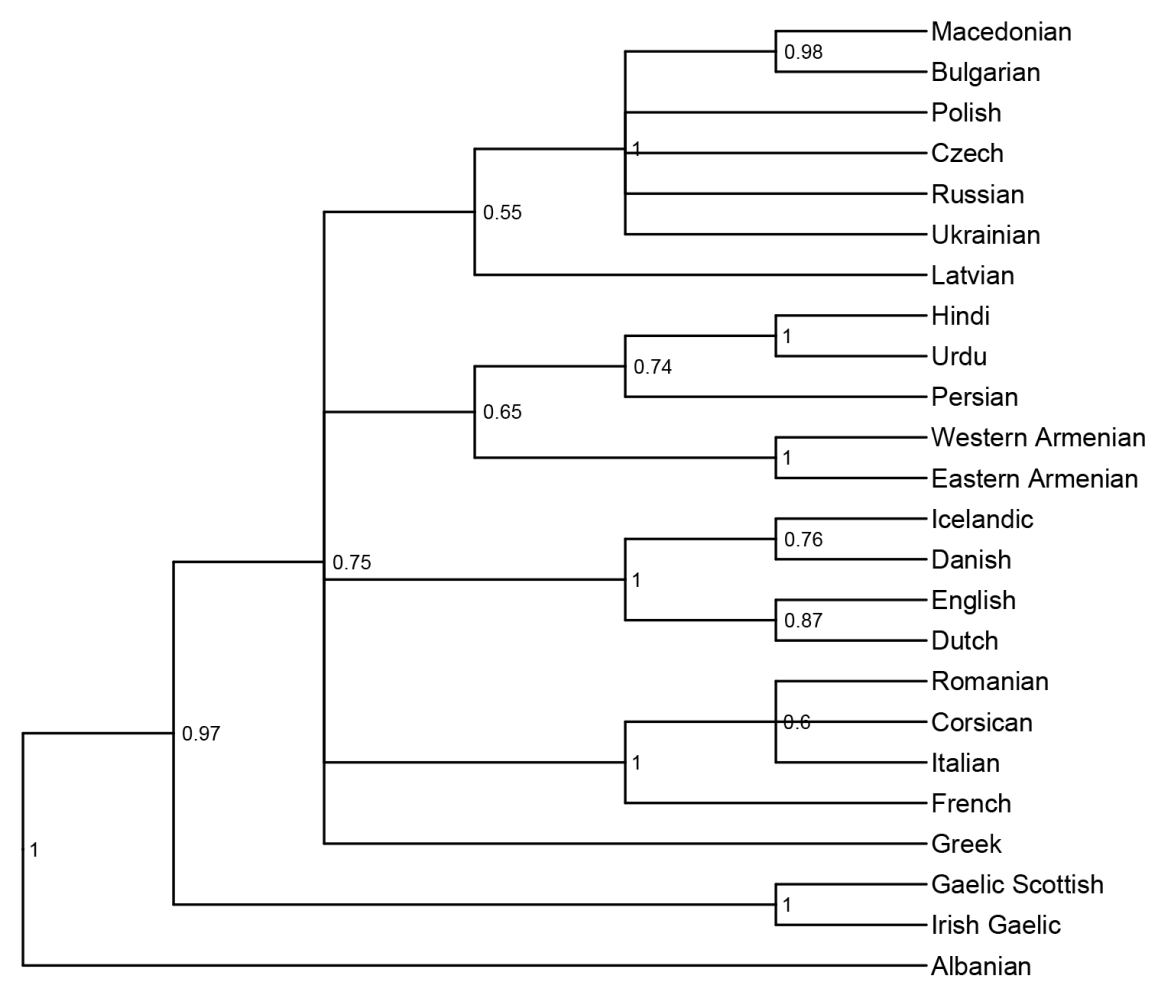
Consensus tree of the *ASJP* language similarity inference. The values at each internal node indicate the posterior clade support of that node.

**Figure 3.**
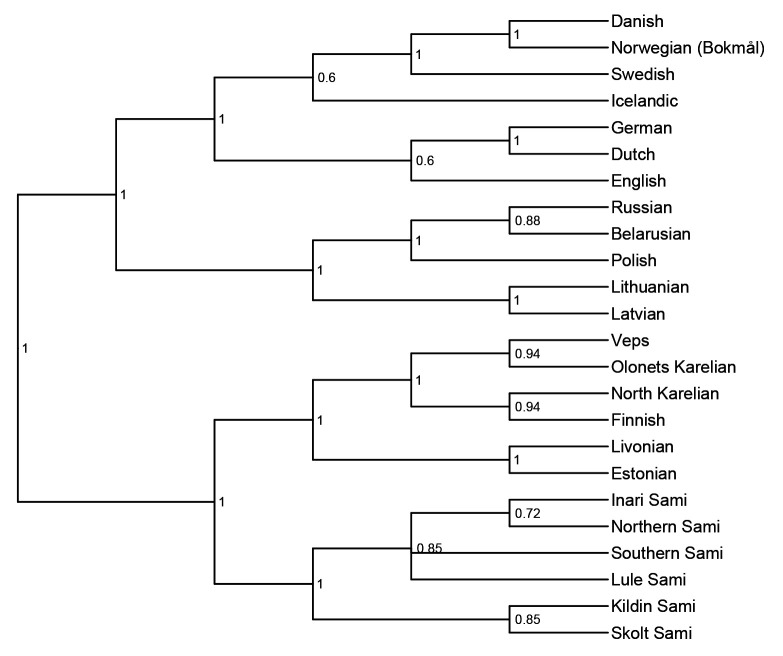
Consensus tree of the *Northeuralex* language similarity inference. The values at each internal node indicate the posterior clade support of that node.

Before we can investigate the model results concerning the accuracy in identifying contact effects, we need to ascertain whether the model succeeds at identifying the major phylogenetic clades. Recall that the goal of this analysis is *not* to infer the correct tree topology of the given languages. Rather, we analyse the tree topologies here solely to check if the model is able to capture the clades well established in earlier research. The consensus trees constructed from the posterior samples of the model show that the model was able to capture the general topology of relationships between the languages in question (see Figure 2 and Figure 3). As reference trees for this comparison, we use the trees in Jäger (2018) and Dellert (2019) for the *ASJP* and *Notheuralex* databases respectively.

The clades the models identifies correctly with high support are Slavic, Indo-Iranian, Romance, Germanic, and Celtic in the *ASJP* dataset, and Germanic (with subdivisions in North and West Germanic), Balto-Slavic, Slavic (with East and West Slavic), Baltic, Uralic (with Finnic and Sámi) in the *Northeuralex* dataset. Further, in the *ASJP* dataset, Albanian is identified as an Indo-European outgroup, as is common in such analyses (cf. Ringe *et al.*, 2002, p. 90). However, the model was not able to retrieve more opaque relationships that are sometimes proposed such as Graeco-Armenian (see Clackson, 1994) or Italo-Celtic (see discussion in Eska, 2009). Or, even if the status as a subgroup is unclear, frequently emerge as subgroups in phylogenetic analyses (e.g. in Ringe *et al.*, (2002) or Chang *et al.*, (2015)).

The level of support that well-established clades exhibit in this model indicates that this inference technique was successful and despite the drawbacks of distance-based methods (see Section *Advantages, biases and limitations of the approach*), the method can be deemed accurate enough to continue analyzing the results.

### Confound strength

**Figure 4.**
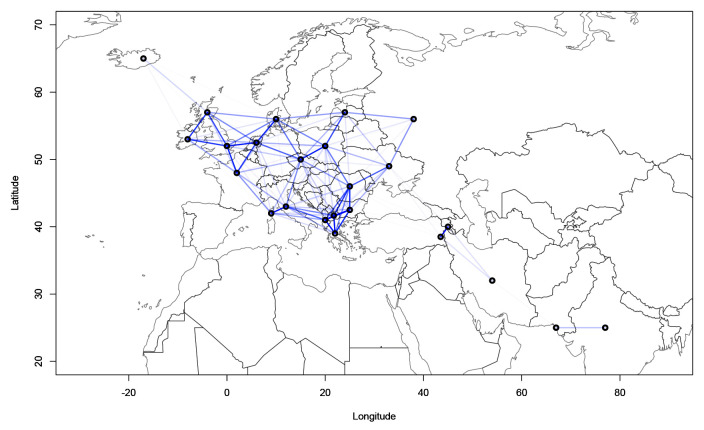
Strength of the geographical confounds between selected languages in the *ASJP* dataset.

**Figure 5.**
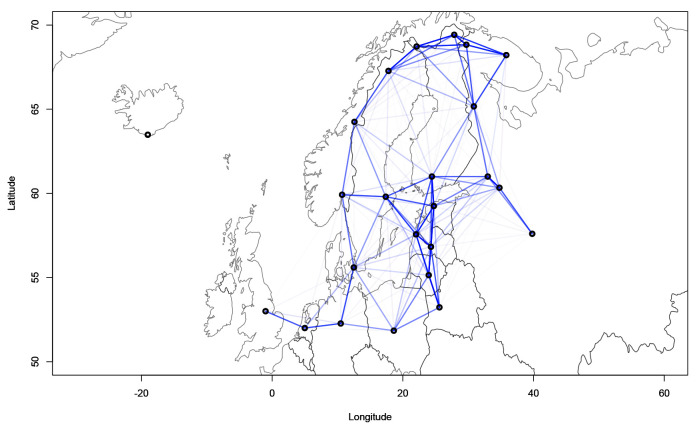
Strength of the geographical confounds between selected languages in the *Northeuralex* dataset.

Figure 4 and Figure 5 show the geographical confound strengths between each language pairing. ^5^ Those strengths are the normalized covariance values of the geographical covariance matrices. The strength line segments in the figures indicate the strength of the geographical confound. From the modelling perspective, the confound strength has to be interpreted as the geographically conditioned similarity of the linguistic data. In other words, if two languages exhibit a strong geographical signal, it means they are in part similar because of the geographical variable. Interpreted from the perspective of phylogenetic similarity, strong geographical covariance between two languages means that they are less similar phylogenetically than they seem, as some of the similarity can be attributed to geographical proximity.

Both maps show that strong geographical covariances are inferred for the Balkan region, northwest Europe, the northeastern Baltic Sea area and northern Scandinavia. This coincides with the previous assumptions about the linguistic contact zones. Further, strong pairwise covariances can be detected for Italian and Corsican, Czech and Polish, and Western and Eastern Armenian. The findings in Figure 5 are compatible with previous computational analyses of contact lines such as Dellert (2019, pp. 262–265) which show strong contact effects especially in the area of the eastern Baltic coastline and northern Scandinavia.

### Clade-level analysis

For the tree-level analysis, we compared the posterior consensus trees of the model with and without the geographical variable. Going forward, the pure phylogenetic model is referred to as the *confounded* model while the *deconfounded* model refers to the model with geographical control.

**Figure 6.**
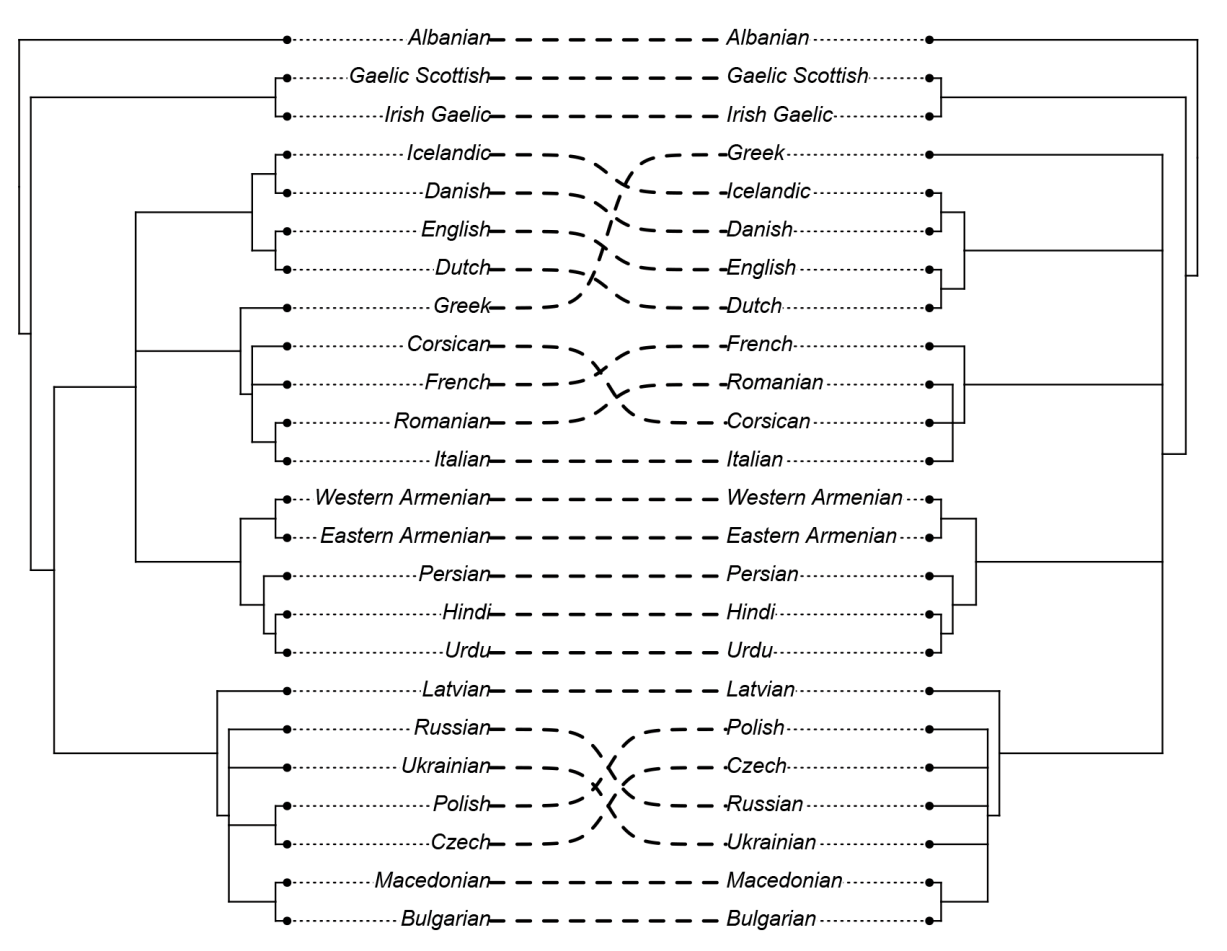
Comparison of the consensus trees of the *ASJP* dataset (created using the R-package *phytools* ( Revell, 2012). *Left*: confounded tree *Right*: deconfounded tree

Firstly, we investigated changes in the consensus trees between the two models. Figure 6 shows a direct comparison between the two consensus trees of the *ASJP* dataset.

In this tree comparison we can observe that Greek was moved from being clustered with Romance to a not clearly determinable outgroup position within Indo-European, a reclassification that is more in line with the consensus view of Greek not being a Romance language. Further, Corsican is then clustered more closely with Italian instead of French.

However, not in line with the consensus view is that in the geographically controlled model, Polish and Czech are no longer clustered together, but are moved to an indeterminate position within Slavic. This points towards the interpretation that the model over-corrects for geospatial effects in this case (see discussion in Section *Advantages, biases and limitations of the approach*).

The tree topologies of the *Northeuralex* dataset do not show differences between the confounded and deconfounded consensus trees. This does not mean that the geographic control did not affect the results, merely that the differences in clade support do not change a clade support from > 0.5 to < 0.5 or vice versa since the consensus trees show a clade whenever support is > 0.5.

As a next step, we compared the differences in tree support for individual clades between the confounded and deconfounded models on both *ASJP* and *Northeuralex* databases. Table 2 and Table 3 show the 10 clades with the strongest differences (both positive and negative). A reduction in clade support from the confounded to the deconfounded model means that adding the geographical control variable causes the model to construe some of the similarities between the constituent languages as contact-induced rather than phylogenetic. Conversely, an increase in support indicates that the clade in question is phylogenetically more plausible based on the geographical analysis.

**Table 2.** Differences in clade support in the *ASJP* database between the confounded and the deconfounded models. Column *change* indicates the change in tree support when switching on the geographical control.

change	difference	clade constituents
0.43 → 0.16	-0.27	Greek, Italian, French, Corsican, Danish, Dutch, English, Icelandic, Romanian
0.60 → 0.39	-0.21	Czech, Polish
0.60 → 0.40	-0.20	Greek, Italian, French, Corsican, Romanian
0.34 → 0.15	-0.20	Italian, French, Romanian
0.92 → 0.74	-0.17	Urdu, Hindi, Persian
0.00 → 0.25	0.25	Latvian, Danish, Dutch, English, Icelandic
0.06 → 0.26	0.20	Urdu, Hindi, Persian, Italian, French, Corsican, Romanian
0.05 → 0.25	0.20	Czech, Polish, Bulgarian, Macedonian
0.44 → 0.60	0.17	Italian, Corsican, Romanian
0.07 → 0.23	0.16	French, Romanian

**Table 3.** Differences in clade support in the *Northeuralex* database between the confounded and the deconfounded models. Column *change* indicates the change in tree support when switching on the geographical control.

change	difference	clade constituents
1.00 → 0.60	-0.40	English, Dutch, German
1.00 → 0.60	-0.40	Icelandic, Norwegian (Bokmål), Danish, Swedish
0.26 → 0.15	-0.11	Inari Sami, Skolt Sami
0.26 → 0.15	-0.11	Inari Sami, Skolt Sami, Kildin Sami
0.26 → 0.15	-0.11	Lule Sami, Southern Sami, Northern Sami
0.72 → 0.85	0.13	Lule Sami, Southern Sami, Northern Sami, Inari Sami
0.74 → 0.85	0.11	Skolt Sami, Kildin Sami
0.33 → 0.41	0.08	Lule Sami, Southern Sami
0.66 → 0.72	0.06	Northern Sami, Inari Sami
0.83 → 0.88	0.05	Belarusian, Russian

It has to be noted, however, that these changes in clade support are not independent. In essence, an increase in clade support for one clade could be caused by a reduction in the competitor clade. For example, the increase in clade support for Italian, Corsican, and Romanian we observe in Table 2 might be caused by the fact that the model downweighs the clade consisting of Italian, French, and Romanian based on inferred contact strength. It is therefore not necessarily the case that these three languages are more likely to constitute a clade based on geography, rather that the alternative hypothesis – Italian, French, and Romanian – has been deemed less likely.

In Table 2, we see that the largest reduction in clade support is in a clade consisting of the Romance and Germanic languages plus Greek which is strongly decreased due to geographical effects. As observed above, Czech and Polish, as well as a clade grouping Greek with the Romance languages, was reduced. Moreover, both the support for Indo-Iranian as well as a clade of Italian, French, and Romanian was decreased. This indicates that some of the similarities between those languages are partly geographically conditioned.

The results from the *Northeuralex* database show many changes pertaining to Sámi, particularly identifying contact networks in Lule Sami, Southern Sami, and Northern Sami and Inari Sami, Skolt Sami, and Kildin Sami. Further, the North Germanic (Icelandic, Norwegian (Bokmål), Danish, and Swedish) and West Germanic (English, Dutch, and German) clades receive less support in the deconfounded model.

### Language-level analysis

In a second step, we retrieved the pairwise phylogenetic distances of both models and compared the changes. Doing this enables the direct comparison of language pairs which decreased/increased their phylogenetic distance due to adding the geographical variable. Table 4 and Table 5 show the top 10 of these these distances for each dataset. ^6^

**Table 4.** Top ten changes in pairwise phylogenetic distance in the *ASJP* dataset between the confounded and deconfounded models.

difference (norm.)	Language pair
-0.34	Macedonian, Albanian
-0.28	Macedonian, Latvian
-0.26	Bulgarian, Albanian
-0.20	Russian, Latvian
-0.19	Czech, Latvian
0.56	Albanian, Greek
0.47	Albanian, Persian
0.46	Albanian, Eastern Armenian
0.36	Corsican, Albanian
0.35	Albanian, Western Armenian

**Table 5.** Top ten changes in pairwise phylogenetic distance in the *Northeuralex* dataset between the confounded and deconfounded models.

difference (norm.)	Language pair
-0.10	North Karelian, Swedish
-0.09	Olonets Karelian, Swedish
-0.07	North Karelian, Norwegian (Bokmål)
-0.07	Finnish, Swedish
-0.04	Veps, Swedish
0.23	Kildin Sami, Swedish
0.21	Polish, Finnish
0.21	Belarusian, Finnish
0.19	Russian, Finnish
0.19	Northern Sami, Danish

In Table 4, we found that, predominantly, distances involving Latvian and Albanian changed the most between the two models: the phylogenetic distance between Albanian and other languages in the Balkan Sprachbund were reduced while distances between other languages were increased. However, the increase in similarity between these languages calls for comment: according to the consensus tree ( Figure 2), Albanian is still an outgroup even under the deconfounded model. The changes in the pairwise distances therefore mean that Albanian has been, relatively speaking, moved away in phylogenetic similarity from the other Balkan Sprachbund languages, which resulted in an inevitable decrease in distance from other languages. As can be seen in the consensus tree, this does not mean that Albanian is therefore particularly close to e.g. Persian, just that a movement away from geographically close languages results in an overall decrease in distance from other languages.

Further, Latvian shows decreased similarity with both western and eastern Slavic languages.

In the *Northeuralex* dataset, we found weaker changes overall; most reduction in phylogenetic similarity is found in pairwise distances between Germanic and Sámi languages on the Scandinavian Peninsula. Conversely, the model reports increases in phylogenetic similarity for Finnish, and some Indo-European languages, which goes against the common consensus. The reason for this result may be that the model overcompensates for Finnish showing geographical effects in the Uralic context by moving it closer to other languages instead.

### Character-level analysis

Recall that the model infers the geographical and phylogenetic distances between languages on the character-level, which means that for every character, the model calculates a corresponding log-likelihood value for the character to be geographically conditioned ( *γ* in the model formula in Section *Model architecture*). High values of this parameter for a certain character indicate that it has a strong geographical conditioning; i.e., the character is likely to be borrowed. If the model succeeds at finding geographical contact patterns, we would expect to find that the actual loanwords we see in the languages in question correspond to high values of *γ* in the model results.

**Figure 7.**
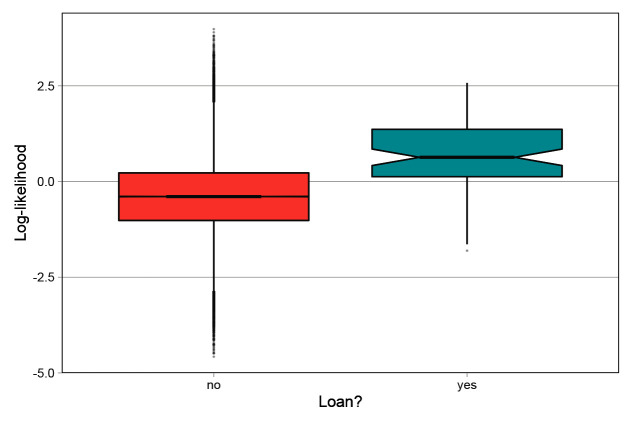
Distribution of per-character geographical log-likelihood by whether or not they are loanwords.

To evaluate the character-level accuracy of the inferences, we relied on the gold-standard loanword dataset provided in the supplements of Dellert (2019), ^7^ which is itself a modification of the World Loanword Database (WOLD) ( Haspelmath & Tadmor, 2009). This enables the direct comparison between the *Northeuralex* dataset model output and the loanword database. For this analysis, we selected all loanwords where the source and target languages are present in the model, which yields a total of 82 borrowed characters. Figure 7 shows the distributions of log-likelihood values for the geographical variable for each character by whether or not they are indicated as loans in Dellert (2019).

The figure shows that the borrowed characters are attributed a larger log-likelihood in the model. To further corroborate the difference between the groups, we ran a univariate Bayesian regression model with standard normal priors using the R-package *brms* ( Bürkner, 2017). In this model, the log-likelihood was the outcome variable and the binary variable of the borrowings was the predictor. The results show a detectable difference between borrowings = ‘yes’ (mean 0.63, 95% CI [0.42, 0.84]) and borrowings = ‘no’ (mean -0.40, 95% CI [-0.41, -0.40]). Therefore we can conclude that the model succeeds in assigning borrowed words a higher log-likelihood. However, the difference is only present in population means. Concretely, although the model assigns borrowings a higher geographical conditioning on the population-level, it fails at clearly identifying individual characters as borrowings.

An accurate classification of characters could therefore not be done. This is crucial for evaluating the usefulness of the model insofar as the model can not be used for identifying loanwords for this reason.

## Advantages, biases and limitations of the approach

After evaluating the results on several levels of analysis, we now turn to a more in-depth scrutiny of the implications and usefulness of the model for phylogenetic research.

### Summary of the analysis

The analysis has shown that the Gaussian process model approach yields mostly accurate inferences of contact zones and their effects on phylogenetic inference. The model succeeds in many respects at deconfounding phylogenetic distances between languages. However, the model seems to overestimate certain confounds especially with languages that are both highly phylogenetically related and geographically close (see discussion in Section *Biases and limitations that reduce the efficacy of the model*).

Overall, however, the method provides insights at different levels of analysis, which is useful to investigate micro-level effects such as pairwise distances as well as macro-level effects on a tree topology or individual clades. Despite this, the granularity is not infinitely scalable as the model cannot be used for loanword classifications (see discussion in section *Character-level analysis*).

Moreover, it has been noticed that due to the strong correlation between reductions and increases in clade support or pairwise language distances, it is not always discernible whether an increase in tree support or language distance for a clade or pair of languages is due to a reduction in another part of the tree.

### Biases and limitations that reduce the efficacy of the model

Several issues limit the accuracy of the approach; some of which are methodological limitations due to how the model is set up, and some of which are biasing factors that interfere with the model’s inferences. This section is intended to sketch out these issues along with some considerations about how to address them.

First of all, the most obvious limitation of this approach is that it relies on a distance-based architecture rather than inferring character evolution as most state-of-the-art phylogenetic models do. This is an issue that cannot be overcome without entirely re-designing the model under an evolutionary model paradigm: the Gaussian processes used in this study are inherently distance-based as they take in information about geographical and phylogenetic relationships in the form of pairwise distances. Related to this point, the model cannot capture historical contact lines as accurately as the geospatial distance matrices used in these analyses are stationary across time can. In effect, this approach does not model changes in contact strength over time as would be possible in a character-evolution model.

A related limitation that encompasses several sub-issues is the inability of singular Gaussian process kernels to handle non-positive-definite matrices. This means that each one of the categories (languages in this case) needs to be proportionally distant from any other category. This means that if there are three languages *A*, *B* and *C*, *A* cannot be at the same time close to *B* but different from *C* without *B* also being different from *C*. Consider the two distance matrices *D*_1_ and *D*_2_.


$$D_{1}=\;\left[\begin{array}{ccc} & 1 & 4\\ 1 & & 3\\ 4 & 3 & \end{array}\;\right]\;D_{2}=\left[\begin{array}{ccc} & 1 & 4\\ 1 & & 1\\ 4 & 1 & \end{array}\;\right]$$

Here, *D*_1_ yields a positive definite matrix outcome of the GP kernel while *D*_2_ does not. The reason for this is that *D*_2_ is not representable in 2D Euclidean space since the distances between the points are not proportional. ^8^

Why is this issue important in linguistic datasets? Geospatial (or, for that matter, all contact-induced) relationships between languages cannot accurately be represented as points in Euclidean space. That is, some languages have several contact points with other languages or nonlinear contact relationships. For example, French is in close contact with both Flemish and Spanish, hence the pairwise distances between French and Flemish and French and Spanish would both be very small while the distances between Flemish and Spanish would be much greater. Further, there can be long-distance or nonlinear contacts such as contacts between English and Spanish in the Americas or French and Arabic in Northern Africa. Additionally, there are contact relationships that are non-geographic, such as Latin influence on several central and western European languages in the middle ages. One could argue, however, that a point-like representation is a sufficiently informative abstraction, yet in such models, we cannot rule out potential mismatches between the Euclidean representations and the in reality observable contacts.

This modeling issue could be addressed, however, by giving each language a covariance matrix of its own that is inferred separately at each step based on a custom distance matrix outfitted with bespoke distances, based on topology and actual contact lines. However, this would increase computation times by a factor equal to the number of languages (i.e. for the approach at hand this would mean a computation time increase of a factor of 25). Even with a moderate number of languages in the dataset (10–30), this would result in computation times of several weeks, even with fully optimized code.

## Conclusion

We have shown in this study that the proposed Gaussian process model can identify areas, clades and language pairs that have been subject to geospatial effects, making them seem genetically closer than they are. This approach succeeds at partially removing those geospatial confounds, thereby mitigating the risk of mistaking contact-induced similarity between languages as support for closer genetic relationships.

While this model is effective overall, we identified some weaknesses that are due to how Gaussian processes in general handle distance matrices and the known issue that languages are represented as point-like units in such models. Further, we found that the character-based loanword identification failed in the model, meaning that the model in its current form cannot be used to identify borrowings in the dataset, as the model accuracy is too low on such a fine-grained level. We identified problems with chain convergence in the model stemming from the Gaussian process setup and the large number of latent parameters. However, these issues can be mitigated by running more chains and averaging over the posterior samples.

## Ethics and consent

Ethical approval and consent were not required

## Data Availability

The datasets that were used in this study are publicly accessible. The similarity judgements dataset (based on Wichmann *et al.*, (2016)) created by Jäger (2018) are accessible at https://osf.io/cufv7/. The data of NorthEuraLex ( Dellert *et al.*, 2020) is available at http://www.sfs.uni-tuebingen.de/~jdellert/pubs/jdellert-diss-supplements.tar.gz. Open Science Framework: Gaussian process models for geographic controls in phylogenetic trees (online appendix) https://doi.org/10.17605/OSF.IO/PK3FX This project contains the following extended data: Appendix.pdf Creative Commons Attribution 4.0 International license (CC-BY 4.0).

## Footnotes

^1^The structure of the data is explained in Section.

^2^In fact, this is true for any real number where *δ* = *λ* .

^3^Publicly accessible at https://osf.io/cufv7/.

^4^Accessible online at https://osf.io/8vw5e.

^5^The same figures can be found in ( Hartmann & Jäger, 2023) without the map data and with language labels.

^6^Note that in order to make the distance matrices comparable, the pairwise distances were normalized such that the largest inferred distance corresponds to 1 in each model before calculating the differences between the models. A change in -0.34 means that from the confounded to the deconfounded model, the pairwise distance between the two languages in question shrunk by 34 percent of the largest phylogenetic distance.

^7^The supplements of Dellert (2019) were downloaded from the following repository: http://www.sfs.uni-tuebingen.de/~jdellert/pubs/jdellert-diss-supplements.tar.gz (last accessed 24.01.2023.

^8^This, however, is only a cursory explanation as the mathematical reason why the posititve definiteness is not fulfilled is grounded in the definition of the Gaussian process kernel.
