# Supplementary material for: Gaussian process models for geographic controls in phylogenetic trees
Source: Open Res Eur. 2024 Jan 22;3:57. Originally published 2023 Apr 17. [Version 2] doi: 10.12688/openreseurope.15490.2 (PMC11110112; doi:10.12688/openreseurope.15490.2)
Supplement: Peer review history

# Reviewer report: Reviewer response for version 2

**Published:** 2025-02-21

**Authors:** Natalia Chousou-Polydouri, Nico Neureiter

**Affiliations:** 1 University of Zurich,, Zurich, Switzerland; 2 Department of Geography, University of Zurich (Ringgold ID: 27217), Zurich, Zurich, Switzerland

The article introduces a method for detecting contact areas, which can also be used to control for contact effects when inferring phylogenetic relationships. The subject of the article is very interesting and it addresses a pressing need in both contact and phylogenetic studies. We find though that the goal of the paper is not clear, and the methods are not sufficiently described and motivated. Unless we are missing a key point, we think that the method’s ability to detect contact areas is fundamentally flawed. At the same time, the method’s utility in improving phylogenetic inference (through correcting for contact effects) is doubtful for a variety of reasons, many of which are already acknowledged in the paper.

 We still want to commend the authors for approaching this important problem in a novel way. Gaussian processes provide a flexible way to explain data probabilistically and they can be naturally generalized to a mixture of multiple processes (inheritance and contact). The fact that they can be implemented in existing probabilistic programming languages like Stan is another advantage. In light of this, we hope that an adaptation of the model can resolve the issues brought up in this review. However, we cannot approve the article in its current form.

**Geographic part of the model - confound strength**

 The most crucial point of our review concerns the geographic part of the model and the calculation of the confound strength. If we are not missing something, the geographic part of the model constrains the covariance matrix G to be a transformation of the geographic distances. Since confound strength values are normalized entries of G, they would also be a scaled version of the geographic distance. Since G and G are fixed across languages,  Gtj=G epx(-G,tjG) is a fixed and continuously decreasing function of G,tj. It is not clear if and how the linguistic data are reflected here. Looking at the maps included in the paper reinforces the interpretation above. Darker edges reflect shorter distances and we could not find a counterexample. 

 If the above understanding of the geographic aspect of the model is correct, it would unfortunately make any interpretation of the confound strength essentially meaningless. Resolving this issue would require changing the model (e.g. estimate G as a latent variable in Stan, and only use geographic distances as a prior).

**Goal of the paper/method**

 It is not clear how the method is proposed to be used. In the introduction, the focus seems to be on improving phylogenetic inference by controlling for contact which is treated as just a confounder. However, later, the focus shifts to detecting contact areas, languages pairs that have participated in contact events and even characters that are plausibly affected by contact.

 As we explained above, we are not convinced that the proposed method can be used to detect contact. It could be argued though that the current methodology could be used to calculate a contact proxy in order to improve phylogenetic inference, in line with the goal of the paper as stated in the beginning. In this case though, the phylogenetic methodology chosen is puzzling. UPGMA, apart from being a distance-based method, performs usually worse than Neighbor-Joining and assumes a strict clock, an assumption that is most often violated in linguistic evolution.

**Description of methods**

 The paper is very short in explaining the methods. The Stan model is mostly clear (some more details and motivation of choices would be useful, see below), but a lot of the analysis actually happens in post processing (UPGMA, consensus tree, computing the confounding strength,...). These post processing aspects are not described fully and required speculation in the review.

 Page 4:

 Is there a reason why a fixed mean of 0 makes sense here? Could you estimate it? A general preference for a certain feature state could be represented by a mean which is non-zero, but constant across languages (i.e. logit(ps,l)=s,l+s,l+s and estimate s as a parameter in Stan).

 You use UPGMA to infer phylogenetic trees from the samples of the covariance matrix P later on, but it seems that in the Stan model P is not constrained to follow the correlation structure implied by a phylogenetic tree. What could be the effect of this? E.g. it seems that any contact effects that are above/below geographic expectations (i.e. distant languages with more contact than expected or proximate languages without contact effects) would be interpreted as closer/more distant relatives in P. 

 What is the motivation for using these particular GP kernels? How are they appropriate for linguistic evolution?

 You are explaining that δP are sampled latent variables, but you don’t mention how you obtain δG. Is it just the geodetic distance between the locations of the languages?

 Page 5:

 It is not entirely clear how the results of Stan are used as input for UPGMA. Is it the case that the P matrix is used as a similarity matrix for UPGMA?

 Page 6:

 It is unclear how the consensus tree was calculated. On page 5, it is described as an MCC tree, while on figures 2 and 3 a majority-rule consensus tree is described. This choice has potential ramifications for the calculation of the average standard deviation of split frequencies when it is calculated for all the clades present in the consensus tree (as different types of consensus trees can include different clades).

 Page 7:

 How are phylogenetic distances defined/calculated? Are you working directly with the δP values here?

 More explanation/motivation is needed for the normalization choice here. The extreme values have a very strong effect on all other values and make a comparison with other values difficult. It’s not entirely clear why normalization is necessary or whether other ways of achieving it were considered.

**Convergence issues**

 The paper’s explanation for the lack of convergence is not convincing to us. The authors describe a scaling factor of the covariance matrix as a possible unidentifiability, which would hinder convergence. However, we do not understand why this scale factor would be unidentifiable: while the scale factor does not affect the tree topology, it does affect the likelihood and the prior probability of the Gaussian process model. Otherwise, the scaling factor only represents a single added dimension in the parameters spaces and we don’t see why it would be particularly difficult for Stan to sample.

 Even though non-convergence of parameters we are not interested in (like the scale factor of the matrix) is indeed not a primary concern, it might be indicative of deeper convergence problems.

 More details should be presented regarding convergence statistics. E.g. The authors mention that they ran four chains but provide only one value for the average standard deviation of split frequencies. On page 14 they mention that they calculated one value per chain, while one needs a pair of chains to calculate this convergence statistic. Additionally, it does not seem that the values of this convergence statistic are sufficiently low (especially the values for clades with higher support than 10% are very high), cf the guidelines of MrBayes recommending the statistic to be lower than 0.01 in order to consider that two runs have converged.

 It seems also that the reported values for the average standard deviation of split frequencies is calculated only for the clades present in the consensus tree (for the low values of the statistic - 0.016 and 0.031), instead of all the splits. This would indicate that only the consensus tree has converged, rather than the whole posterior distribution. Even the higher values of the statistic are not calculated using all splits, leading us to suspect that the real value is even higher. Unfortunately the simulation study did not seem to provide any clear insights into the convergence issues.

**Minor points**

 Page 3: 

 Model architecture: Describing M as "the statistical model" and including it as a node in the graphical model seems odd. Usually the nodes represent variables and the whole graph is a description of the model. 

 The figure should just be this:        G → D ← P

 Typo: simulatenous → simultaneous 

 Page 10:

 Typo: that themodel infers the the geographical –> that the model infers the geographical

 Page 14:

 Typo: randomtree –> random tree

 Typo: majoritysupported –> majority-supported

If any, are all the source data and materials underlying the results available?

Yes

Is the work original in terms of material and argument?

Yes

Is the work clearly and cogently presented?

Partly

Is the argument persuasive and supported by evidence?

No

Does the research article contribute to the cultural, historical, social understanding of the field?

Yes

Does it sufficiently engage with relevant methodologies and secondary literature on the topic?

Yes

Reviewer Expertise:

phylogenetic methods, bayesian statistics, inference of linguistic contact and linguistic areas, linguistic evolution

We confirm that we have read this submission and believe that we have an appropriate level of expertise to state that we do not consider it to be of an acceptable scientific standard, for reasons outlined above.

# Reviewer report: Reviewer response for version 2

**DOI:** 10.21956/openreseurope.18448.r40296

**Published:** 2024-06-03

**Authors:** Grégoire Clarté

**Affiliations:** 1 Mathematics, The University of Edinburgh, Edinburgh, Scotland, UK

The article uses UPGMA method to reconstruct phylogenetic trees. The article is interesting in that it tries to include the possibility of lateral transfer explained in terms of geographical proximity.

 The first remark, which is not mentionned in the article if I read correctly, is that the UPGMA method is criticable for building phylogenies. Although this method allow to compute a "distance" the process of building the tree with this distance is dubious (why middle point for the ancestor of two languages? Other questions arise in the model itself). It seems this model leads to strange results in the experiments, for example the position of Greek in Fig 6, which is not discussed. For more discussion on the subject there are articles for example Steel, M., Hendy, M. D., & Penny, D. (1988) ‘Loss of information in genetic distances.’ on the use of distances, and Greenhill "Language Phylogenies: Modelling the Evolution of Language". Overall, I would say that the model presented lacks interpretability and support from the process usually used to model linguistic evolutions.

 The model itself is a little strange, it seems quite distant from the usual modelling in linguistics. It is not entirely clear how the cognates are used. Does it correspond to the "features" in the model? That is, can a cognate appear several times in the same language and different languages? I think this model is very far from reality, and can lead to strange results when the number of cognates increase (with the model, we would expect the cognates to have independent presence/anbsence in unrelated far away languages, say Sapmi languages and Germanic). The data from Northeuralex is even more strange, as it mixes several families which are known to be unrelated. Does this induce bias in the analysis? Are the cognates from e.g. Estonian that are actually borrowed from Latvian meaningful? I think independent studies would have been advisable. As a side note: the use of the Northeuralex data involves a computation of cognates using automated tools. There were warnings on this subject by List if I remember well.

 Concerning the use of Stan, the analysis of the output is an interesting addition (and useful).

 I think the model is interesting but further modelling assumptions should be discussed in the article. Additional comparison with other methods could be interesting as well (for example other methods only based on phylogenetic signal, such as the Dollo model). Nevertheless, it is interesting to see both geographical and evolutionary parameters learnt jointly.

If any, are all the source data and materials underlying the results available?

Yes

Is the work original in terms of material and argument?

Yes

Is the work clearly and cogently presented?

Yes

Is the argument persuasive and supported by evidence?

Partly

Does the research article contribute to the cultural, historical, social understanding of the field?

Partly

Does it sufficiently engage with relevant methodologies and secondary literature on the topic?

Partly

Reviewer Expertise:

Bayesian phylolinguistic, Bayesian modelling, Numerical methods.

I confirm that I have read this submission and believe that I have an appropriate level of expertise to confirm that it is of an acceptable scientific standard, however I have significant reservations, as outlined above.

# Reviewer report: Reviewer response for version 2

**DOI:** 10.21956/openreseurope.18448.r40298

**Published:** 2024-05-20

**Authors:** Frederic Blum

**Affiliations:** 1 Max Planck Institute for Evolutionary Anthropology, Leipzig,, Saxonia, Germany; 2 Chair for Multilingual Computational Linguistics, University of Passau, Passau, Bavaria, Germany

The paper contributes a workflow of using Gaussian Processes for controlling for spatial biases during the inference of phylogenetic trees in linguistics. Based on two case studies, the authors show how the Gaussian Processes can identify contact between language pairs and its implications on the branch structure of the inferred trees. The method is a useful contribution, since spatial bias was often discussed, but rarely addressed quantitatively in phylolinguistic studies (cf. Neureiter et al. 2022). Considering the limitations (which the authors make explicit), the method will need to be verified on additional datasets in the future. That being said, typological studies should find a way for controlling for spatial and genealogical biases, and the present paper adds another important technique to the typologists toolbox.

 The paper provides a clear line of argumentation that relates to the relevant previous research. One limitation that is not (yet) discussed in the paper is the focus on two datasets that consist mostly of European languages. It will be interesting to see how the method applies to other contact areas. Another possible comparison would be to test this method directly against similar approaches, such as Neureiter et al's (2022) contacTrees. It is likely that future phylogenetic studies adopt one of either method to implement such a spatial control, and the present study succeeds in highlighting this importance very well. Future phylogenetic studies should use some of the available methods to implement such a spatial control, and the study succeeds in highlighting this importance very well.

 The authors could improve on two points. First, it is not clear to me how the distance measure between languages was computed. In case I have simply missed this, it should probably be highlighted a bit more, but it seems like this has only been made explicit for the simulated data of the Section 6.3 appendix. Considering that different ways exist to compare distances between languages (topographic, geodesic, etc), the processing of distances should be made explicit. This includes the source of the coordinates for each language (assuming a single-point location was used, e.g. Glottolog coordinates, or the individual datasets), and the publication of any pre-processing scripts for computing the distances, along the other scripts. Since one of the authors is also part of another paper that relates to linguistic geopgrahy and distance measures [Refer ref 1], this should be reasonable to elaborate on.

 Second, some further improvements could be made with respect to the presentation of the data and code. Please make the code fully reproducible.

 - Since the data is not part of the versionized repository, the download link might fail to work in the future. Given that the data itself is quite small, it would be recommendable to add the data to the repository, so that it gets a DOI.

 - Please upload the fitted models to some repository like OSF. Since the model takes long to run, it is easier for the reviewer and audience to reproduce the results if the model files are provided from the start.

 - It would have been super useful to have a small Readme.md that introduces the individual files, introducing the dependencies between individual scripts.

 - Since R packages are notoriously difficult to versionize, it is recommendable to use a tool like `groundhog` that stores the package versions and enables their installing. Without this, the code might break in the future.

 - Hard-coded paths needed to be changed in order to run the scripts locally. Using relative paths and `paste` would ensure reproducibility across platforms.

 - The code did not run without fixing individual lines, e.g. the unzip-command had to be changed in order to correctly read the file, and for the Stan-model the download only referred to the metadata. Alternatively, provide a How-To for the download in the Readme file.

 ```bash

 > untar("jdellert-diss-supplements.tar.gz",files="inferred-cognates.tsv")

 tar: inferred-cognates.tsv: Not found in archive

 tar: Error exit delayed from previous errors.

 Warning message:

 In untar("jdellert-diss-supplements.tar.gz", files = "inferred-cognates.tsv") :

 ‘/usr/bin/tar -xf 'jdellert-diss-supplements.tar.gz' 'inferred-cognates.tsv'’ returned error code 1

 ```

 Small typos:

 - Page 4 (Section 3)> 'Fromboth'

 - Page 10 (Section 4.5): 'themodel'

 - Page 11 (Section 4.5): 'Rpackage'

 - Page 12 (Section 5.2): 'are stationary across time can.'

If any, are all the source data and materials underlying the results available?

Partly

Is the work original in terms of material and argument?

Yes

Is the work clearly and cogently presented?

Yes

Is the argument persuasive and supported by evidence?

Yes

Does the research article contribute to the cultural, historical, social understanding of the field?

Yes

Does it sufficiently engage with relevant methodologies and secondary literature on the topic?

Yes

Reviewer Expertise:

Bayesian statistics; computational modeling of linguistic data

I confirm that I have read this submission and believe that I have an appropriate level of expertise to confirm that it is of an acceptable scientific standard, however I have significant reservations, as outlined above.

## References

[1] : Euclide, the crow, the wolf and the pedestrian: distance metrics for linguistic typology. *Open Research Europe* .2023;3: 10.12688/openreseurope.16141.1 10.12688/openreseurope.16141.1 38989155 PMC11234076

# Reviewer report: Reviewer response for version 2

**DOI:** 10.21956/openreseurope.18448.r37655

**Published:** 2024-01-25

**Authors:** Thomas Brochhagen

**Affiliations:** 1 Universitat Pompeu Fabra, Barcelona, Catalonia, Spain

The authors have convincingly addressed the concerns I raised in my earlier assessment. I thank them for the  informative investigation of the convergence issues and for sharing their code.

If any, are all the source data and materials underlying the results available?

Partly

Is the work original in terms of material and argument?

Yes

Is the work clearly and cogently presented?

Yes

Is the argument persuasive and supported by evidence?

Yes

Does the research article contribute to the cultural, historical, social understanding of the field?

Yes

Does it sufficiently engage with relevant methodologies and secondary literature on the topic?

Yes

Reviewer Expertise:

Computational cognitive modelling; Bayesian methods; lexical typology

I confirm that I have read this submission and believe that I have an appropriate level of expertise to confirm that it is of an acceptable scientific standard.

# Reviewer report: Reviewer response for version 1

**DOI:** 10.21956/openreseurope.16746.r33777

**Published:** 2023-07-20

**Authors:** Thomas Brochhagen

**Affiliations:** 1 Universitat Pompeu Fabra, Barcelona, Catalonia, Spain

The article "Gaussian process models for geographic controls in phylogenetic trees" introduces a new approach to phylogenetic inference that factors in potential geographic biases, which may otherwise skew the inference (e.g., contact zones that explain why linguistic features are shared add noise to phylogenetic inference that may mistake this commonality as signal of common ancestry). Geographic bias is a common problem in phylogenetic inference, and a central topic in typological research more generally. The proposal is a Gaussian process model that infers latent phylogenetic similarity after accounting for observed geographic similarity (closeness of point estimates of where a language is spoken). The model is evaluated on two datasets where geographic influences have already been studied so that model predictions can be benchmarked against past research. Results suggests that the model succeeds in characterizing geographic bias, as reflected by inferred phylogenetic similarities; which is to say that, as desired, geographic confounds are factored out of the inference.

 The model has a few limitations. These include its failure to make accurate fine-grained predictions about individual linguistic features with respect to geographic bias; and chain convergence issues by the sampler. That being said, issues and limitations are clearly highlighted and discussed. They do not detract from the article's main contributions but rather contextualize them.

 This is a very clear and well-executed study. The idea of using Gaussian processes to model geographic biases is becoming well-established, and adding them as a component of phylogenetic inference makes a lot of sense, as also demonstrated by the results. The evaluation on the two datasets, and additional checks on loanword identification, convincingly show that the proposed model is also of practical import; as well as where its limitations are. It would be interesting to see if the model scales to larger datasets, and to which extent divergences between a model that does not factor out potential geographic bias and one that does can be used to explore heretofore less prominently researched contact areas. Overall, I think this is a nice and solid contribution that will surely be further stress-tested in future research.

 I have two main comments to offer. The first has to do with chain convergence (Results section). The authors mention that, with this model, chain convergence is not guaranteed. The reasoning offered makes sense to me, but I think that this methodological deviation from the standard diagnostics (in this case, checking that the same tree topology is expressed instead of computing, say, split R-hat as one would do normally) warrants further discussion and experimentation in the appendix so that researchers that want to use this approach know whether to trust their estimates or not. This brings me to my second and more important comment: the (re-)usability of the methods. As far as I can see, the Stan implementation is not shared. I would highly encourage the authors to make it openly-available so that the approach can easily be put to use and evaluated by others. Auxiliary scripts used to, e.g., compare the tree topologies obtained from different chains would also be useful to have. While I think the first issue can also be tackled in future research, I think the second (code availability) should be addressed in the current version. This is why I went for "Approval with Reservations" instead of "Approval"

**Minor comments:**

   * Footnote 1 is incomplete (the section number is missing);

   * "Stan" is named after Stanislaw Ulam. It's conventionally written with only the first letter capitalized.

If any, are all the source data and materials underlying the results available?

Partly

Is the work original in terms of material and argument?

Yes

Is the work clearly and cogently presented?

Yes

Is the argument persuasive and supported by evidence?

Yes

Does the research article contribute to the cultural, historical, social understanding of the field?

Yes

Does it sufficiently engage with relevant methodologies and secondary literature on the topic?

Yes

Reviewer Expertise:

Computational cognitive modelling; Bayesian methods; lexical typology

I confirm that I have read this submission and believe that I have an appropriate level of expertise to confirm that it is of an acceptable scientific standard, however I have significant reservations, as outlined above.

# Response

**Published:** 2023-12-27

**Authors:** Frederik Hartmann

**Affiliations:** University of North Texas, USA

We thank the reviewer very much for their helpful comments and we attempted to make the paper and our arguments clearer following the reviewer’s suggestions. The main changes are: (1) publishing the source code on Zenodo (see link in the paper body) and (2) providing a detailed investigation into the convergence issues and how they affect clade credibility (see online Appendix).   Specific responses to individual points:   I have two main comments to offer. The first has to do with chain convergence (Results section). The authors mention that, with this model, chain convergence is not guaranteed. The reasoning offered makes sense to me, but I think that this methodological deviation from the standard diagnostics (in this case, checking that the same tree topology is expressed instead of computing, say, split R-hat as one would do normally) warrants further discussion and experimentation in the appendix so that researchers that want to use this approach know whether to trust their estimates or not. > We ran additional tests on simulated data to be able to better pinpoint the cause of the convergence issues. The appendix (Section 6.3) now contains a detailed discussion of those tests and their results.   This brings me to my second and more important comment: the (re-)usability of the methods. As far as I can see, the Stan implementation is not shared. I would highly encourage the authors to make it openly-available so that the approach can easily be put to use and evaluated by others. Auxiliary scripts used to, e.g., compare the tree topologies obtained from different chains would also be useful to have. While I think the first issue can also be tackled in future research, I think the second (code availability) should be addressed in the current version. This is why I went for "Approval with Reservations" instead of "Approval"  > The code files have now been uploaded to the repository as indicated in the paper (10.5281/zenodo.10247032).   Minor comments:     * Footnote 1 is incomplete (the section number is missing);     * "Stan" is named after Stanislaw Ulam. It's conventionally written with only the first letter capitalized. > These errors were corrected

# Reviewer report: Reviewer response for version 1

**DOI:** 10.21956/openreseurope.16746.r32026

**Published:** 2023-06-20

**Authors:** Tiago Tresoldi

**Affiliations:** 1 Uppsala University, Uppsala, Sweden

The paper significantly contributes to computational historical linguistics ("phylogenetic linguistics") addressing a key challenge in the field, as is very welcomed. The authors present a novel approach for modelling language change not exclusively as vertical transmission with random mutations, considering the significant role of horizontal transmission via language contact which is promoted by geographic proximity. They propose a hybrid approach combining spatial with phylogenetic modelling to address this. The probability of a language having a particular feature is assumed to depend on both the presence or absence of the feature in the language's spatial neighbours and in its genetic relatives. The paper then tests this model on datasets of known language families with known contact effects.

 The analysis results show that the Gaussian process model approach yields mostly accurate inferences of contact zones and their effects on phylogenetic inference, yielding "deconfounded" trees. Note, however, that the model also appears to overestimate certain confounds, especially with languages that are both highly phylogenetically related and geographically close. The authors acknowledge several limitations of the approach, including its reliance on a distance-based architecture and the inability to use it for loanword classifications. Despite these limitations, the paper concludes that the method provides valuable insights at different levels of analysis, which is useful for investigating micro-level effects such as pairwise distances and macro-level effects on a tree topology or individual clades.

 The results should be interpreted as an initial exploration in this direction. While it would have been beneficial to evaluate the performance on other datasets, including synthetic ones, or to allow their system to infer the geographical similarity matrix 'G' just as `P`, these are not limitations that detract from the value of the paper. The authors' transparency in highlighting the biases and limitations of their approach is commendable. The results presented in the "Clade-level analysis" and "character-level analysis" sections are auspicious, and it seems evident that they are considering the generalization of the method with additional matrices, such as one for "cultural power" (addressing issues such as those of English of French).

 Simultaneously, the paper introduces a methodology that deviates from the conventional approach in the field, which typically involves following tutorials on BEAST2. The authors' use of STAN and their construction of UPGMA trees for the posterior is refreshing, providing diversity in the field's methodology. However, the impact of this approach is diminished by the need for public access to the software pipeline, making it challenging for the reviewer to assess the method and reproduce the results. The source for the G matrix is not provided, and the contents of Figure 1 and the "Methods" session are too limited to a re-implementation aiming for reproducing results. It is hoped that the authors plan to release and refine the software pipeline post-publication. Furthermore, without access to the source code and all data, it is challenging to investigate potential issues related to the 'G' matrix and its subproducts, such as the apparent lack of linkage between Persian and Urdu/Hindi, as indicated in Figure 4. The availability of the software pipeline as part of the submission would significantly enhance the paper's value. While the decision to require the complete source code and all data ultimately lies with the editor, it is strongly recommended that the authors consider releasing it.

**Specific issues:**

- Figure 2 could be improved by indicating the support level used to accept branches. It appears to be 0.5, but confirmation would be helpful.
- In Figure 3, it would be beneficial to highlight that we are ultimately examining a cladogram. For instance, the Indo-European and Uralic members could be emphasized.
- The discussion of Greaco-Armenian and Italo-Celtic could benefit from citing more recent sources, such as Olander et al. 2022's "The Indo-European Language Family: A Phylogenetic Perspective".
- The first mention of "phylogenies" or "phylogenetic linguistics", as well as "cognate", could be explained for readers less familiar with the field. This is particularly relevant as we are discussing "cognates" as "root-meaning pairs" (i.e., more as in computational historical linguistics than traditional comparative linguistics).
- The statement "A well-known potential problem of phylogenetic..." could use a citation for support.
- The claim that "language contact almost always happens under spatial proximity" should be backed up with a citation.
- The statement "The probability is calculated as the logit sum of the variables ρ and γ." would benefit from a prior definition of what ρ and γ model.
- The repetition of "investigate" in "we investigate the strength of the geographical confound between languages on a general level, before investigating" could be avoided for better readability.
- The sentence "The clades the models identifies correctly..." could be restructured for clarity. For instance, "The models correctly identify several clades with high support. In the ASJP dataset, these include Slavic, Indo-Iranian, Romance, Germanic, and Celtic. In the Northeuralex dataset, they include Germanic (subdivided into North and West Germanic), Balto-Slavic, Slavic (split into East and West Slavic), Baltic, and Uralic (further divided into Finnic and Sámi)."
- The sentence "The level of support that well-established clades exhibit in this model indicates that this inference technique was successful and despite the drawbacks of distance-based methods (see Section Advantages, biases and limitations of the approach), the method can be deemed accurate enough to continue analyzing the results." should be split into two sentences for better readability.
- "This points towards the interpretation that the model over-corrects for geospatial effects in this case." -- I would say something like "This suggests that the model might be over-correcting for geospatial effects in this instance."
- "A reduction in clade support from the confounded to the deconfounded model means that adding the geographical control variable causes the model to construe some of the similarities between the constituent languages as contact-induced rather than phylogenetic." -- I would say something like "A reduction in clade support when transitioning from the confounded to the deconfounded model indicates that adding the geographical control variable leads the model to interpret some of the similarities among the constituent languages as contact-induced rather than phylogenetic."
- The sentence "Table 4 and Table 5 show the top 10 of these these distances for each dataset." has a repeated "these". A better phrasing could be "Table 4 and Table 5 display the top 10 distances for each dataset, demonstrating the most significant changes."
- There appear to be some formatting issues with the citations, such as nested parentheses in "(Greenhill et al., 2009)", "Chang et al. (2015))", "(Hartmann & Jäger, 2023)", and the missing parenthesis at the end of footnote 7. These should be corrected.

If any, are all the source data and materials underlying the results available?

Partly

Is the work original in terms of material and argument?

Yes

Is the work clearly and cogently presented?

Yes

Is the argument persuasive and supported by evidence?

Yes

Does the research article contribute to the cultural, historical, social understanding of the field?

Yes

Does it sufficiently engage with relevant methodologies and secondary literature on the topic?

Yes

Reviewer Expertise:

I have been doing research in computational historical linguistics and have interested in modelling geographic controls in phylogenies.

I confirm that I have read this submission and believe that I have an appropriate level of expertise to confirm that it is of an acceptable scientific standard, however I have significant reservations, as outlined above.

## References

[1] The Indo-European Language Family.2022; 10.1017/9781108758666 10.1017/9781108758666

# Response

**Published:** 2023-12-27

**Authors:** Frederik Hartmann

**Affiliations:** University of North Texas, USA

We thank the reviewer very much for their helpful comments and we attempted to make the paper and our arguments clearer following the reviewer’s suggestions. The main changes are: (1) adding references and (2) publishing the source code on Zenodo (see link in the paper body).   Specific responses to individual points:   The availability of the software pipeline as part of the submission would significantly enhance the paper's value. While the decision to require the complete source code and all data ultimately lies with the editor, it is strongly recommended that the authors consider releasing it. > The code and data have been shared on Zenodo    Figure 2 could be improved by indicating the support level used to accept branches. It appears to be 0.5, but confirmation would be helpful.  > The threshold of 0.5 is now indicated in the figure caption.   In Figure 3, it would be beneficial to highlight that we are ultimately examining a cladogram. For instance, the Indo-European and Uralic members could be emphasized. > It is now indicated that this is a cladogram of Indo-European and Uralic languages.     The discussion of Greaco-Armenian and Italo-Celtic could benefit from citing more recent sources, such as Olander et al. 2022's "The Indo-European Language Family: A Phylogenetic Perspective". > The references were updated according to the reviewer’s suggestions.   The first mention of "phylogenies" or "phylogenetic linguistics", as well as "cognate", could be explained for readers less familiar with the field. This is particularly relevant as we are discussing "cognates" as "root-meaning pairs" (i.e., more as in computational historical linguistics than traditional comparative linguistics). > After the first mention of “phylogenies”, the subsequent text makes it sufficiently clear, in our opinion, what it means. We further added a footnote explaining our usage of “cognate”.   The statement "A well-known potential problem of phylogenetic..." could use a citation for support. > A citation to this effect has been added.     The claim that "language contact almost always happens under spatial proximity" should be backed up with a citation. > We did not find a suitable quotation, so we added a footnote justifying the claim.   The statement "The probability is calculated as the logit sum of the variables ρ and γ." would benefit from a prior definition of what ρ and γ model. > The paragraph was restructured and clarifications were added.   The repetition of "investigate" in "we investigate the strength of the geographical confound between languages on a general level, before investigating" could be avoided for better readability. > The sentence was changed accordingly.     The sentence "The clades the models identifies correctly..." could be restructured for clarity. For instance, "The models correctly identify several clades with high support. In the ASJP dataset, these include Slavic, Indo-Iranian, Romance, Germanic, and Celtic. In the Northeuralex dataset, they include Germanic (subdivided into North and West Germanic), Balto-Slavic, Slavic (split into East and West Slavic), Baltic, and Uralic (further divided into Finnic and Sámi)." > The sentence was changed accordingly.     The sentence "The level of support that well-established clades exhibit in this model indicates that this inference technique was successful and despite the drawbacks of distance-based methods (see Section Advantages, biases and limitations of the approach), the method can be deemed accurate enough to continue analyzing the results." should be split into two sentences for better readability. > The sentence was changed accordingly.     "This points towards the interpretation that the model over-corrects for geospatial effects in this case." -- I would say something like "This suggests that the model might be over-correcting for geospatial effects in this instance." > The sentence was changed accordingly.     "A reduction in clade support from the confounded to the deconfounded model means that adding the geographical control variable causes the model to construe some of the similarities between the constituent languages as contact-induced rather than phylogenetic." -- I would say something like "A reduction in clade support when transitioning from the confounded to the deconfounded model indicates that adding the geographical control variable leads the model to interpret some of the similarities among the constituent languages as contact-induced rather than phylogenetic." > The sentence was changed accordingly.     The sentence "Table 4 and Table 5 show the top 10 of these these distances for each dataset." has a repeated "these". A better phrasing could be "Table 4 and Table 5 display the top 10 distances for each dataset, demonstrating the most significant changes." > The sentence was changed accordingly.     There appear to be some formatting issues with the citations, such as nested parentheses in "(Greenhill et al., 2009)", "Chang et al. (2015))", "(Hartmann & Jäger, 2023)", and the missing parenthesis at the end of footnote 7. These should be corrected. > These errors were corrected, provided the typeset version reflects our changes.
